# The effect of free-breathing on left ventricular rotational mechanics in normal subjects and patients with duchenne muscular dystrophy

**DOI:** 10.1186/1532-429X-17-S1-Q22

**Published:** 2015-02-03

**Authors:** Meral Reyhan, Zhe Wang, Hyun J Kim, Nancy Halnon, Paul J Finn, Daniel B Ennis

**Affiliations:** 1 Bioengineering, University of California Los Angeles, Los Angeles, CA, USA; 2 Radiological Sciences, University of California Los Angeles, Los Angeles, CA, USA; 3 Biomedical Physics Interdepartmental Program, University of California Los Angeles, Los Angeles, CA, USA; 4 Pediatric Cardiology, University of California Los Angeles, Los Angeles, CA, USA

## Background

Patients with Duchenne Muscular Dystrophy (DMD) develop respiratory impairment and signs and symptoms of cardiac involvement at an early age. Quantitative measures of cardiac function from MRI tagging can be used to stage disease progression and monitor the response to therapy. Traditional cardiac MRI exams, however, require repeated breath holding, which places a notable burden on patients with DMD and can impart intrathoracic pressure changes that alter cardiac function. The effect of free-breathing on quantitative measure of left ventricular (LV) rotational mechanics for DMD patients is incompletely understood. *Objective*: To evaluate differences in LV rotational mechanics acquired during breath holding (BH), free-breathing with averaging (AVG), and free-breathing with respiratory bellows gating (BEL).

## Methods

16 healthy volunteers (1 female, 27.8±3.9 years) and 5 DMD patients (all male, 11.3±3.7 years) were scanned on a 3.0T Siemens Scanner with BH, AVG and BEL. ORI-SPAMM (Reyhan. M et. al. JMRI 2014;39(2):339-345) was used to acquire short-axis images at the base and apex of the heart. 280-330x280-330mm field-of-view, 6mm slice thickness, 192x192 acquisition matrix, 395 Hz/pixel receiver bandwidth, TE/TR =2.33-2.39/4.71-4.83 ms, 8 phase encode lines per segment, 12° imaging flip angle, 8 mm tag spacing, GRAPPA 2. LV twist and circumferential-longitudinal shear (CL-shear) angle were calculated using the Fourier Analysis of STimulated echoes (FAST) method (Reyhan. M et. al. JMRI 2012;35(3):587-593).

## Results

**Figure 1:**
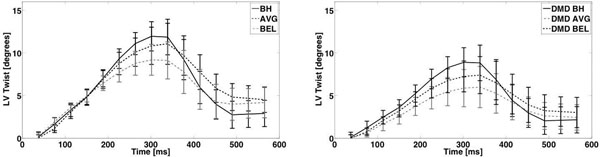
Mean LV twist of (A) healthy volunteers and (B) patients with DMD for breath-held (BH, solid), free-breathing with averaging (AVG, dashed), and bellows gated (BEL, dash-dotted) methods. BH LV twist is generally higher compared to AVG or BEL, especially at its peak.

Figure 1 demonstrates the mean LV twist curve from healthy volunteers (A) and DMD patients (B). Errors bars represent the standard deviation of each group. Detailed results are reported in Table 1. Significant intra-group differences in peak LV twist were found between BH and BEL using Wilcoxon signed rank test for normal (p=0.003) and DMD (p=0.004). Both apical and basal epicardial radii show significant intra-group differences between the BH and BEL (p=0.007 and p=0.006). No significant difference was detected between healthy volunteers and DMD patients for peak CL-shear angle.

**Table 1:** 

	Normal			DMD		
	
	BH	AVG	BEL	BH	AVG	BEL
Mean Peak LV Twist [deg]	12.9±2.3	11.3±3.8	10.2±3.6*	10.5±3.6	9.3±3.4	8.6±3.6*

Mean Peak CL-Shear Angle [deg]	6.4±1.7	6.2±1.4	5.9±1.6	5.9±1.7	5.7±1.4	5.6±2.1

LV Apical Epicardial Radius [mm]	23.3±2.5	25.0±3.1	25.9±3.4*	17.1±3.1	18.4±3.5	20.2±4.6*

LV Basal Epicardial Radius [mm]	32.3±2.3	32.9±2.4	33.1±2.5*	27.0±3.0	28.7±3.3	30.4±4.1*

* Indicates statistical difference from BH

## Conclusions

The observed increase in end systolic epicardial radius during BEL compared to BH indicates that afterload is increased which accords with a decrease in twist. The decrease in rotation combined with the increase in end-systolic radii cause CL-shear angle to pseudo-normalize. Overall, a free breathing strategy is a preferred method for patients with limited breath hold ability due to the non-negligible difference between BH and BEL results. However, for longitudinal studies where breath-hold conditions cannot be controlled CL-shear angle may be a better measure of rotational mechanics.

## Funding

AHA 13BGIA14530010 to Daniel B. Ennis and AHA 11PRE6080005 to Meral L. Reyhan

